# Genetic influences of the intercellular adhesion molecule 1 (ICAM-1) gene polymorphisms in development of Type 1 diabetes and diabetic nephropathy

**DOI:** 10.1111/j.1464-5491.2006.01948.x

**Published:** 2006-10

**Authors:** J Ma, A Möllsten, M Prázny, H Falhammar, K Brismar, G Dahlquist, S Efendic, H F Gu

**Affiliations:** Diabetes Center Karolinska (DCK), Department of Molecular Medicine and Surgery, Karolinska University Hospital, Karolinska Institute Stockholm, Sweden; * Department of Clinical Sciences, Pediatrics, Umeå University Umeå, Sweden; † Third Department of Internal Medicine, First Faculty of Medicine, Charles University Prague, Czech Republic

**Keywords:** genetic association, intercellular adhesion molecule 1, nephropathy, single nucleotide polymorphism, Type 1 diabetes mellitus

## Abstract

**Aim:**

The intercellular adhesion molecule-1 (ICAM-1) gene is located on chromosome 19p13, which is linked to Type 1 diabetes (T1D). ICAM-1 expression is related to development of T1D and diabetic nephropathy. The present study aims to evaluate the genetic influence of ICAM-1 gene polymorphisms on the development of T1D and diabetic nephropathy.

**Methods:**

Five valid single nucleotide polymorphisms (SNPs) were genotyped in 432 T1D patients (196 patients had diabetic nephropathy) and 187 non-diabetic control subjects by using dynamic allele-specific hybridization (DASH) and pyrosequencing.

**Results:**

SNPs rs281432(C/G) and rs5498 E469K(A/G) had high heterozygous indexes. They were significantly associated with T1D [*P* = 0.026, OR = 1.644 (95% CI 1.138–2.376) and *P* < 0.001, OR = 2.456 (1.588–3.8)]. Frequencies of the C allele in SNP rs281432(C/G) and the A allele in SNP rs5498 E469K(A/G) increased stepwise from non-diabetic control subjects to T1D patients without diabetic nephropathy and T1D patients with diabetic nephropathy. Further analysis for these two SNPs indicated that T1D patients had increased frequency of the common haplotype C-A, in comparison with non-diabetic control subjects (38.1 vs. 32.1%, *P* = 0.035).

**Conclusion:**

The present study provided evidence that SNPs rs281432(C/G) and rs5498 E469K(A/G) in the ICAM-1 gene confer susceptibility to the development of T1D and might also be associated with diabetic nephropathy in Swedish Caucasians.

## Introduction

Type 1 diabetes mellitus (T1D; OMIM 222100) is an autoimmune disease. The destruction of insulin-secreting β-cells of the islets of Langerhans leads to hyperglycaemia, which is a key factor in the development of diabetic microvascular complications such as retinopathy, nephropathy and neuropathy. The development of T1D and diabetic complications is strongly modulated by genetic and environmental factors [1]. Identification of the genes predisposing for T1D and its specific microvascular pathology and dissection of genotypic and phenotypic interactions are of importance for better understanding of the pathogenesis of T1D and mechanism of vascular damage.

To date, several loci conferring susceptibility to T1D have been identified, i.e. the human leucocyte antigen complex in chromosome 6p21.3, the insulin gene in 11p15.5 and the cytotoxic T lymphocyte-associated antigen-4 (CTLA4) gene in 2q33.2 [2]. Furthermore, genome-wide scans have predicted that T1D susceptibility genes may reside at other chromosomal regions, including chromosome 19p13 [2–4]. The intercellular adhesion molecule (ICAM-1) gene is located in this chromosomal region. ICAM-1 is a 90-kDa cell surface glycoprotein belonging to the immunoglobulin superfamily and it is involved in the firm attachment of leucocytes to endothelium [5,6]. It is normally expressed at low levels on the surface of arterial endothelial cells. ICAM-1 mRNA and protein of ICAM-1 were reported to be increased by hyperglycaemia [7,8]. In experimental studies using T1D animal models, increased ICAM-1 expression accompanies progression of T1D [9] and also diabetic nephropathy [10,11]. Inhibition of ICAM-1 expression in non-obese diabetic mice prevents or delays the onset of T1D [12]. Clinical studies indicate that the level of plasma adhesion molecule is elevated in T1D patients [13,14]. Furthermore, it has been proposed that the increased levels of plasma adhesion molecule may be a sign of pre-clinical T1D in children [15]. Therefore, the ICAM-1 gene represents a strong positional and biological candidate for the susceptibility to the development of T1D and diabetic nephropathy.

In the last 7 years, several genetic association studies for the ICAM-1 gene in T1D have been published. All these previous studies were designed for analysis of one or two single nucleotide polymorphisms (SNPs) in the ICAM-1 gene, and conflicting results were reported. Guja *et al*. first demonstrated that the transmission of the G allele of SNP K469E(A/G) was increased in Romanian T1D families [16]. Nishimura *et al*. found this SNP to be associated with adult-onset T1D patients in Japanese subjects [17]. However, the association of this SNP with T1D was not found in Danish, Finnish and British Caucasians [18,19]. Furthermore, Nejentsev *et al*. reported that another non-synonymous SNP G241R(G/A) in the ICAM-1 gene was associated with T1D. The G241 allele was highly transmitted in T1D families of Finnish, British, Romanian, European and American Caucasian origin [20]. The aim of the present study was to determine the genetic influence of the ICAM-1 gene polymorphisms in the development of T1D and diabetic nephropathy. We have therefore conducted a comprehensive genetic association study for five SNPs in the ICAM-1 gene, including the previously studied SNPs, i.e. K469E(A/G) and G241R(G/A). All 432 T1D patients and 187 non-diabetic control subjects who participated in the study were Swedish Caucasians. Of the T1D patients, 196 had diabetic nephropathy.

## Patients and methods

### Subjects

A group of 432 (women 234/men 198) patients with T1D were included in the present study. The patients were diagnosed according to the World Health Organization criteria (WHO 1985 [21]). All patients with T1D had urinary albumin excretion rate (UAER) measured in at least two consecutive overnight urine samples. One hundred and ninety-six (103/93) T1D patients had diabetic nephropathy (UAER ≥ 20 µg/min); 187 (116/71) non-diabetic healthy individuals, with no family history of diabetes, served as control subjects. All T1D patients and non-diabetic control subjects were Swedish Caucasians. Clinical characteristics of all subjects are summarized in Table 1. Informed consent was obtained from all subjects and the study was approved by the local ethics committees. Genomic DNA was extracted from peripheral blood using a Puregene DNA purification kit (Gentra, Minneapolis, MN, USA).

**Table 1:** Clinical characteristics of T1D patients and non-diabetic control subjects

	Non-diabetic controls	All T1D patients	T1D without diabetic nephropathy	T1D with diabetic nephropathy
*n* (M/F)	187 (71/116)	432 (198/234)	236 (105/131)	196 (93/103)
Age (years)	48 ± 5	45 ± 12	44 ± 12	46 ± 12
Duration (years)	—	31 ± 11	29 ± 10	34 ± 12
BMI (kg/m^2^)	23.0 ± 1.82	25.3 ± 3.44	24.6 ± 3.08	25.8 ± 3.65
SBP (mmHg)	118 ± 14	132 ± 19	126 ± 15	138 ± 21
DBP (mmHg)	73 ± 9	76 ± 9	73 ± 8	78 ± 10
HbA_1c_ (%)	—	7.2 ± 1.25	7.0 ± 1.15	7.4 ± 1.35

All data, mean ± sd.BMI, body mass index; DBP, diastolic blood pressure; SBP, systolic blood pressure.

### SNPs selection and validation

All SNPs in the ICAM-1 gene selected for the present study are recorded in the public dbSNP database. The SNP ID numbers and detailed sequence information are publicly available (http://www.ncbi.nlm.nih.gov/SNP/). Eight SNPs were selected for study. For each SNP, 32 DNA samples from Swedish Caucasians were used to test the frequency of the polymorphisms. The allele frequencies of three SNPs, i.e. rs5490, rs5030348 and rs5495(M56K), which are located, respectively, in the 5′ untranslated region, intron 1 and exon 5, were lower than 1% in our population. These three SNPs were thus excluded and the remaining five SNPs were selected for further genotyping experiments in the larger set of samples.

### SNP genotyping and sequencing analysis

We have used a high-throughput SNP scoring technique called dynamic allele-specific hybridization (DASH [22]). PCR-DASH assays were designed for genotyping all SNPs and the sequence information for all primers and probes designed are given in Table 2. To confirm DASH genotyping data for SNPs rs281432(C/G) and rs5498 E469K(A/G), which were found to be associated with T1D, we have used another high-throughput genotyping method, i.e. pyrosequencing [23] with the same primers as used in the DASH assays.

**Table 2:** The primers and probes used in PCR-DASH assays for the ICAM-1 gene genotyping

SNP ID	Oligo name	Sequence
rs5491	ICAM1–09F	5′- TGACATGCAGtACCTCCTGTGACC-3′
	ICAM1b10R	5′-Biotin-TTAGGCAACGGGGTCTCTATGTCC-3′
	ICAM1–11paRox	5′-CCAGCCCAAGTTGTTGG-Rox-3′
rs281432	ICAM1–13F	5′-CTTGGGGTGATAGGGAGTCATG-3′
	ICAM1b14R	5′-Biotin-CCTTCAGCTATCTAATCCCTGG-3′
	ICAM1–15pcRox	5′-GAGGGTTTCTGAGCAGG-Rox-3′
rs5492	ICAM1b17F	5′-Biotin-GTGGTGCTGCTCaGTGGGGAGA-3′
	ICAM1–18R	5′-CACATCTGGCTCCCGTTTCAGCT-3′
	ICAM1–19pcRox	5′-TTCAGCTCCTTCTCCCCA-Rox-3′
rs1799969	ICAM1–21F	5′-GGGGACCATGGTCTGTTCACTGGA-3′
	ICAM1b22R	5′-Biotin-ACCTGGTCCTCCGAGACTGTGAACA-3′
	ICAM1–23pgRox	5′-CACTGGACGGGCTGTTCAC-Rox-3′
rs5498	ICAM1–29F	5′-AGGAGCACTCAAtGGGAGGTCAtCCG-3′
	ICAM1b30R	5′-Biotin-ACTCACAGAaCACATTCACGGTaACC-3′
	ICAM1–31pgRox	5′-TCAtCCGCGAGGTtACC-Rox-3′

To further examine whether ‘duplicons’ were involved in the ICAM-1 gene, the genomic sequences around SNPs rs281432(C/G) and rs5498 E469K(A/G) were PCR-amplified. The sizes of the PCR-amplified fragments are 419 and 511 bp, respectively, for SNPs rs281432(C/G) and rs5498 E469K(A/G). The direct sequencing analysis using Big Dye Terminator Cycle Sequencing Ready Reaction Kit (Applied Biosystems, Foster City, CA, USA, ABI, model 377 genetic analyser, Perkin-Elmer, Shetton, CT, USA) was performed. The sequences of the primers and optimized PCR conditions are available on request.

### Bio-informatics

Description of the positions of all SNPs was accorded to the genomic contig sequences (accession no. NT_011295). The sequence alignment analysis for all SNPs and the designed assays in human genome was performed using BLAST. The possible structures of PCR products for DASH genotyping experiments were detected with mFold (version 3.1; http://www.bioinfo.rpi.edu/applications/mfold/old/dna/).

### Statistical analyses

Deviation from the Hardy–Weinberg equilibrium for alleles in each SNP was assessed using the appropriate Chi-square distributed statistics. For differences between T1D patients and non-diabetic control subjects, two models were tested comparing either allele frequencies in 2 × 2 contingency tables or genotypes in 3 × 2 contingency tables. Tests for association between genotypes and quantitative traits were performed using Kruskal–Wallis analysis of ranks for traits with non-normal distributions, or alternatively, anova for normally distributed traits. For association estimation, odds ratios (OR) and 95% confidence intervals (CI) were estimated with unconditional logistic regression models between case and control (T1D patients and non-diabetic individuals) groups. All statistical analyses were performed using the BioMedical Data Program (BMDP) version 1.12 and/or STATISTICA version 7.0. Linkage disequilibrium (LD) was carried out using our own algorithms and Arlequin program (http://lgb.unige.ch/arlequin/ in *r*^2^ and/or D′). Heterogeneity in haplotype frequency distribution between T1D and control groups was tested by calculating the test statistic 2*(log-likelihood_T1D_ + log-likelihood_Control_ − log-likelihood_Combined_), which has a χ^2^ distribution with *n*-1 degrees of freedom (*n* is the number of haplotypes).

## Results

The allele frequencies of five SNPs in the ICAM-1 gene are given in Table 3. No significant differences in the allele frequencies were found between the groups of T1D patients and non-diabetic control subjects. However, there were significant differences of genotype distribution in SNPs rs281432(C/G) (*P =* 0.026) and rs5498 E469K(A/G) (*P <* 0.001) between all T1D patients and non-diabetic control subjects (Table 3). The comparison analysis for CC + CG vs. GG in SNP rs281432(C/G) between T1D patients and non-diabetic control subjects indicated that this SNP was associated with T1D [OR = 1.644 (95% CI 1.138–2.376), *P* = 0.008)]. In SNP rs5498 E469(A/G), similar results were found, suggesting that the SNP was associated with T1D [OR = 2.456 (1.588–3.800), *P* = 0.001 for AA + AG vs. GG]. There was no significant difference in genotype distributions of SNPs rs5491(A/T), rs5492(C/G) and rs1799969 R241G(A/G) between the case and control groups studied.

**Table 3:** Allele frequencies of SNPs in the ICAM-1 gene

SNP ID	Location	Contig position*	IUB DNA code	Group	Alleles		Allele frequency	*P*-value
rs5491	Exon 2	1648342	K56M	ND	T 368	A 4	T0.989/A0.011	NS
			W = A/T	T1D	T 893	A 5	T0.994/A0.006	
rs281432	Intron 2	1653460	Intronic	ND	G 224	C 150	G0.599/C0.401	0.069
			S = C/G	T1D	G 467	C 393	G0.543/C0.457	
rs5492	Exon 3	1657092	N155K	ND	G 369	C 3	G0.992/C0.008	NS
			S = C/G	T1D	G 851	C 11	G0.987/C0.013	
rs1799969	Exon 4	1657594	R241G	ND	G 320	A 52	G0.860/A0.140	NS
			R *=* A/G	T1D	G 718	A 136	G0.841/A0.159	
rs5498	Exon 6	1658485	E469K	ND	A 191	G 183	A0.511/G0.489	NS
			R *=* A/G	T1D	A 481	G 379	A0.559/G0.441	

* Contig accession no. NT_011295.

ND, non-diabetic control subjects; NS, not significant.

We found that SNP rs281432(C/G) and rs5498 E469K(A/G) had high heterozygous indexes, although the genotype distributions of SNPs studied in non-diabetic individuals were in the Hardy–Weinberg equilibrium. We thus performed and confirmed the genotyping experiments by using DASH and pyrosequencing. Data using both techniques were matched, and no indication of genotyping error was found. To ascertain whether ‘duplicons’, which might cause high heterozygous indexes, were involved in these two SNPs, direct sequencing analysis by using forward and reverse primers was performed, but no ‘duplicons’ were found.

**Figure 1:**
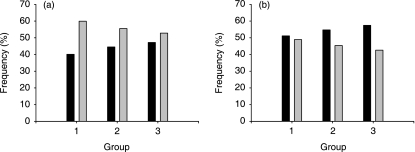
Allele distribution of SNPs (a) rs281432(C/G) and (b) rs5498 E469K(A/G). Group 1, non-diabetic control subjects; group 2, T1D patients without diabetic nephropathy; group 3, T1D patients with diabetic nephropathy; (a) ▪, allele C; ░, allele G; (b) ▪, allele A; ░, allele G.

We attempted to detect possible association of SNPs rs281432(C/G) and rs5498 E469K(A/G) with diabetic nephropathy, although no significant difference of genotype distribution in these two SNPs between the groups of T1D patients with diabetic nephropathy and the patients without diabetic nephropathy was found (Table 4). We dissected the allele frequencies for these two SNPs in non-diabetic control subjects, T1D patients without nephropathy and T1D patients with nephropathy. Results indicated that frequencies of the C allele in SNP rs281432(C/G) and the A allele of SNP rs5498 E469K(A/G) increased gradually from non-diabetic control subjects (40.1 and 51.1%, respectively), to T1D patients without diabetic nephropathy (44.5 and 54.7%), and to T1D patients with diabetic nephropathy (47.2 and 57.4%; Figs 1a and b).

**Table 4:** Genotype distributions of SNPs rs281432 and rs5498

SNP ID	Groups	Genotypes			*P*-value	OR (95% CI)	*P*-value
		CC	CG	GG		CC + CG vs. GG	CC + CG vs. GG
rs281432	Non-diabetic control subjects	30	90	67			
	All T1D patients	72	249	109	0.026*	1.644 (1.138–2.376)	0.008*
	T1D without diabetic nephropathy	35	139	61			
	T1D with diabetic nephropathy	37	110	48	0.529†	1.074 (0.693–1.663)	0.750†
		AA	AG	GG		AA + AG vs. GG	AA + AG vs. GG
rs5498	Non-diabetic control subjects	52	87	48			
	All T1D patients	104	273	53	< 0.001*	2.456 (1.588–3.800)	0.001*
	T1D without diabetic nephropathy	50	156	28			
	T1D with diabetic nephropathy	54	117	25	0.279†	1.076 (0.605–1.914)	0.804†

P-value:

* All T1D patients vs. non-diabetic control subjects;

† T1D patients with nephropathy vs. T1D patients without nephropathy.

To further search for evidence that ICAM-1 gene polymorphisms influence the development of T1D, the degree of LD for all SNPs was examined. Data indicated that the ICAM-1 gene had a low LD (Table 5) and the average probability of haplotype interference across the dataset was moderate. However, a relatively strong LD (|*D*′| = ∼0.7) appeared to extend over the region between SNPs rs281432(C/G) and rs5498 E469K(A/G). Frequencies of haplotypes constructed by these two SNPs in T1D patients and non-diabetic control subjects are summarized in Table 6. Four common haplotype (with at least 5% frequency) including C-A, C-G, G-G and G-A were examined. The haplotype C-A frequency was significantly higher in T1D patients than in non-diabetic control subjects (38.1 vs. 32.1%, *P* = 0.035).

**Table 5:** Pair-wise LD values for SNPs in the ICAM-1 gene

	|*D*′|				
SNPs	rs5491	rs281432	rs5492	rs1799969	rs5498
rs5491	—	1.000	1.000	0.076	0.374
rs281432	0.003	—	0.616	0.127	0.689
rs5492	0.775	0.009	—	0.325	0.621
rs1799969	0.932	0.034	0.004	—	0.296
rs5498	0.363	< 0.001	0.018	0.001	—
	*P*-value				

**Table 6:** The frequencies of haplotypes constructed by SNPs rs281432(C/G) and rs5498 E469K(A/G)

Haplotypes	Non-diabetic control subjects	All T1D patients	*P*-value
C-A	0.321	0.381	0.035
C-G	0.080	0.074	NS
G-G	0.409	0.363	NS
G-A	0.190	0.173	NS

## Discussion

We have carried out a genetic association study of five SNPs in the ICAM-1 gene in Swedish T1D patients and non-diabetic control subjects with a high-throughput SNP scoring technique called DASH. The results indicated that SNPs rs281432(C/G) and rs5498 E469K(A/G) were significantly associated with the development of T1D. Interestingly, these two SNPs had a high heterozygous index in the genotype distribution. We thus confirmed the genotyping experiments by using pyrosequencing. Segmental duplications (duplicons) with > 90% similarity between copies comprise at least 5% of the human genome, which may cause specific allelic and genotypic diversities, such as a high heterozygous index in complex diseases [24,25]. In order to ascertain whether the duplicons are involved in the regions around the SNPs studied, we performed direct sequencing analysis for the subjects with the heterozygous genotypes and found that no duplicon was involved. In the previous genetic association studies of SNP rs5498 E469K(A/G) in T1D, the information concerning genotype distribution of this SNP was unclear [18–20]. We therefore communicated with the authors of previous reports. The summary of genetic association study of this SNP in different ethnic populations in the previous and present studies (Table 7) indicates that the phenomenon of high heterozygous index of this SNP exists in all populations studied. The high heterozygous index may cause a ‘type 1 error’ in genetic-association analysis. Indeed, no significant difference of the allele frequencies of this SNP between T1D patients and non-diabetic control subjects was observed in the present study. By comparison analysis of genotype distributions, however, we were able to detect association of the polymorphisms with T1D. Based on the findings in the present study and the previous reports [16,17], we conclude that the heterozygous genotypes of SNPs rs281432(C/G) and rs5498 E469K(A/G) in the ICAM-1 gene may confer disease risk.

**Table 7:** Comparison analysis of SNP rs5498 E469K(A/G) genotype distribution in four ethnic populations

	Non-diabetic control subjects (%)				T1D patients (%)				
			
Population	*n*	AA	AG	GG	*n*	AA	AG	GG	Reference
Swedish	187	27.8	46.5	25.7	432	24.2	63.5	12.3	The present study
Danish	461	31.7	49.4	18.9	252	27.4	56.7	15.9	Kristiansen *et al*. 2000 [19]
Finnish	212	24.1	50.0	25.9	218	26.6	47.2	26.1	Nejentsev *et al*. 2000 [18]
Japanese	171	12.3	50.9	36.8	164	18.3	47.0	34.7	Nishmura *et al*. 2000 [17]

Rs5498 E469K(A/G) is a non-synonymous SNP and resides in the fifth immunoglobulin-like domain of ICAM-1. To date, no report indicates that this domain is involved in ligand binding of ICAM-1. However, this domain may play a role in an immunodominant epitope of B lymphocytes and dendritic cells [26]. A recent study has demonstrated that this SNP affects ICAM-1 mRNA splicing pattern and TPA-induced apoptosis [27]. In accordance with genetic association studies of this SNP in the present and previous reports [16,17], we suggest that SNP rs5498 E469K(A/G) has a genetic and biological influence related to the pathogenesis of T1D.

Single marker analysis is a useful tool for genetic association studies. Multiplex marker analysis, however, may be more powerful for detecting an association in complex diseases. All previous genetic association reports of the ICAM-1 gene in T1D studied only one or two SNPs, which may reduce the possibility of detecting an association. In the present study, we genotyped five SNPs along the ICAM-1 gene and performed a haplotype analysis based upon population-specific LD. We found that SNP rs281432(C/G) and rs5498 E469K(A/G) might exist in an LD block. The common haplotype C-A of these two SNPs was found to be significantly associated with T1D in Swedish Caucasians. Previously, Nejentsev *et al*. found that SNP rs1799969 R241G(A/G) had an increased transmission rate in T1D patients [20]. This non-synonymous SNP is located between SNPs rs281432(C/G) and rs5498 E469K(A/G), which were not found to be associated with T1D in our population, most likely because of specific population LD diversities.

Biological experiments suggest that genetic variation of the ICAM-1 gene might be involved in the development of diabetic nephropathy [10,11]. To test this hypothesis, we have dissected frequency and distribution of the alleles in SNPs rs281432(C/G) and rs5498 E469K(A/G) among non-diabetic control subjects, T1D patients without nephropathy and T1D patients with nephropathy. Frequencies of the C allele of SNP rs281432(C/G) and the A allele of SNP rs5498 E469K(A/G) increased gradually, suggesting that most likely these alleles confer risk in the developmental process of T1D and also diabetic nephropathy. However, the differences in the allele frequencies and genotype distributions did not reach statistical significance when comparing T1D patients with and without diabetic nephropathy. The sample size is one of the critical components for a successful genetic-association study. The sample size for T1D with nephropathy in the present study and also in the previous reports [17–19] was relatively limited as a result of the difficulty of sample collection.

In conclusion, the C allele of SNP rs281432(C/G) and the A allele of SNP rs5498 E469K(A/G) in the ICAM-1 gene are significantly associated with T1D in Swedish Caucasians. These two alleles confer an increased risk for the development of T1D and might also be associated with susceptibility to diabetic nephropathy.

## Competing interests

None declared.

## Abbreviations

DASH: dynamic allele-specific hybridization
ICAM-1: intercellular adhesion molecule-1
LD: linkage disequilibrium
SNP: single nucleotide polymorphism
T1D: Type 1 diabetes
